# Impact of Bond–Slip Models on Debonding Behavior in Strengthened RC Slabs Using Recycled Waste Fishing Net Sheets

**DOI:** 10.3390/polym16213093

**Published:** 2024-11-01

**Authors:** Huy Q. Nguyen, Taek Hee Han, Jun Kil Park, Jung J. Kim

**Affiliations:** 1 Department of Civil Engineering, Kyungnam University, Changwon-si 51767, Republic of Korea; nguyenquochuy@muce.edu.vn; 2 Faculty of Infrastructure Engineering–Technology, Mientrung University of Civil Engineering, Tuy Hoa 620000, Vietnam; 3 Ocean Space Development and Energy Research Department, Korea Institute of Ocean Science and Technology, 385 Haeyang-ro, Yeongdo-gu, Busan 49111, Republic of Korea; taekheehan@kiost.ac.kr (T.H.H.); jkpark@kiost.ac.kr (J.K.P.)

**Keywords:** recycled WFN, bond–slip law, debonding failure, RC slab, strengthening

## Abstract

This study investigated the performance of recycled waste fishing net sheets (WSs) as a sustainable strengthening material for reinforced concrete (RC) slabs. The primary challenge addressed is the debonding failure caused by the low bond strength at the WS-to-concrete interface. To analyze this, two full-scale RC slabs—one with and one without strengthening—were cast and tested under a four-point bending setup. Finite element (FE) models incorporating existing bond–slip laws were developed using the ABAQUS software to simulate the strengthened slab’s behavior. A sensitivity analysis was performed to assess the impact of bond–slip parameters on the failure mechanism. Experimental results indicated that the WS-strengthened slab enhanced the RC slab capacities by 15% in yield load and 13% in initial stiffness. Furthermore, the maximum shear stress of 0.5τ_max_ or interfacial fracture energy of 0.2G_f_, compared to values proposed by Monti et al., enabled the simulation of the global response observed in the experiment.

## 1. Introduction

Marine debris, especially plastic, poses a serious threat, disrupting aquatic ecosystems and adversely affecting human life [1,2,3,4]. Sources include tourism litter, stormwater debris, ship waste dumping, lost cargo, and discarded fishing gear [5]. Prior estimates suggested that discarded fishing gear comprise about 10% of global marine litter [6,7,8], with annual discard and losses ranging from 500,000 to 1 million tons [9]. Recent research also reveals a troubling fact: nearly 2% of all fishing nets are lost in the ocean annually [10,11]. Made from synthetic materials such as high-density polyethylene (HDPE), polyethylene terephthalate (PET), polyvinyl alcohol (PVA), polyethylene (PE), and polypropylene (PP) [4,12,13], discarded fishing nets pose a high threat to marine ecosystems. These neutrally buoyant nets entangle and endanger aquatic life while disrupting active fishing gear [3,14]. More concerning, microplastics can enter human bodies via the food chain as marine creatures ingest small plastic pieces from the aquatic environment [3,4,15]. Thus, reducing plastic pollution by recycling waste fishing nets (WFNs) is essential for safeguarding marine ecosystems, biodiversity, and human health [16].

Recycling WFNs has effectively protected aquatic ecosystems and provided economic benefits, with roughly 152 tons recovered from the ocean annually [17]. WFNs are upcycled into valuable resources, becoming carpet tiles, components for furniture and luggage, tool handles, and even electronic parts [18]. WFNs are also recycled into filament for 3D printing to manufacture fashionable accessories, recreational products, sporting goods, and fishing tools [17]. Additionally, recycled WFNs have expanded into construction materials, with their applications reaching diverse uses [16,19,20].

Using WFNs as reinforcement fibers in civil engineering applications for concrete, mortar, and soil is a promising development [18]. Kim et al. [21] found that adding WFN fibers (WFs) into lightweight soil enhanced its compressive strength up to 2.5 times. Spadea et al. [22] revealed that incorporating recycled nylon fiber from WFNs into mortar significantly improved the toughness, ductility, and tensile strength (up to 35%). Nguyen et al. [13] demonstrated that WF provided benefits in crack control, enhancing the post-cracking behavior, and transforming concrete from brittle to quasi-brittle. Truong et al. [16,19] showed that using WFN textiles and fibers, reinforced with anchorage methods, can effectively strengthen or retrofit existing concrete structures. While WF reinforcement could improve some mechanical properties [18,23], it also introduced flaws in the mortar matrix, slightly reducing the elastic modulus, durability, and compressive strength [20]. Preparing WF from WFNs and achieving a uniform distribution in mortar cement proved challenging due to the laborious and time-consuming [20] processes involved. Consequently, developing new solutions to expand the practical application scope of recycled WFNs is essential.

To meet such demands, a practical, suitable way to recycle WFNs into sheets that strengthen existing reinforced concrete (RC) structures has been developed by the Korea Institute of Ocean Science and Technology (KIOST). WFN sheets (WSs) hold promise for repairing and rehabilitating coastal structures due to their lightweight nature, corrosion resistance, and high durability, making them well-suited for these demanding environments [24]. Their application resembles well-known fiber-reinforced polymer (FRP) composites, targeting the tensile zones of RC beams, slabs, or columns [25,26]. Nevertheless, some limitations require consideration. WFNs exhibit significantly lower tensile strength than traditional FRP materials made of glass, aramid, carbon, or basalt fibers [19,20,27,28,29]. Another limitation is premature debonding, a common challenge in external bonding techniques, which prevents the strengthened structures using FRP/WS from reaching their load-carrying capacity [30,31]. Debonding failures are complex due to multiple factors at the FRP–concrete interface, like cracks and stress distribution [32,33]. While evaluating the ability of WSs to restore the bearing capacity of RC slabs is crucial, a deeper understanding of the debonding mechanism is essential to unlock their full potential. It will be key to maximizing the effectiveness of WSs in practical applications. To address this need, the impact of bond–slip laws on the debonding failure of flexurally strengthened RC slabs with recycled WSs is examined. A cohesive approach is employed to model the bond behavior between concrete and WS, facilitating the prediction of structural responses [34,35]. Investigating the magnitude and distribution of shear stress at the WS–concrete interface can offer insights into debonding failure mechanisms [36,37]. Nevertheless, the presence of cracks makes predicting the concrete stress distribution challenging. Conversely, the load–deflection curve, easily obtained through experiments, is less sensitive to the locations and sizes of cracks. In addition, the finite element method (FEM) provides a more economical alternative for predicting interfacial stresses and load–deflection response [38]. Using the ABAQUS 2024 software, a finite element (FE) model of the strengthened slab can analyze the bond between WS and concrete [39]. This combined approach with experiments refines design equations and reduces testing time and cost [40].

This study further investigated the response of a strengthened RC slab using a recycled WS, with a specific focus on understanding debonding failure. The performance of the recycled WS in enhancing the load-carrying capacity was evaluated through a full-scale experimental test. Furthermore, an FE model inputting existing bond–slip models was developed to assess their influence on the debonding failure of the strengthened slab. Major characteristic parameters impacting the behavior of strengthened slabs, especially debonding failure, were determined and discussed through sensitivity analysis.

## 2. Materials and Methods

### 2.1. Materials

#### 2.1.1. Concrete and Steel Bar

Table 1 provides the weight ratios of the ingredients used in the concrete mixture for RC slabs, featuring coarse aggregate with a maximum diameter of 20 mm and fine aggregate with a diameter of less than 2.5 mm. The concrete’s compressive strength, recorded at 30 MPa, was determined by testing cylindrical specimens with a diameter of 100 mm and a height of 200 mm, following ASTM C39 standards [41]. All steel bars were hot-rolled ribbed bars with a yield stress of 400 MPa and modulus of elasticity of 200 GPa according to the “Tensile Test Method for Metallic Materials” outlined in KS B 0802 [42].

#### 2.1.2. WFN Sheet and Epoxy Resin

**Figure 1:**
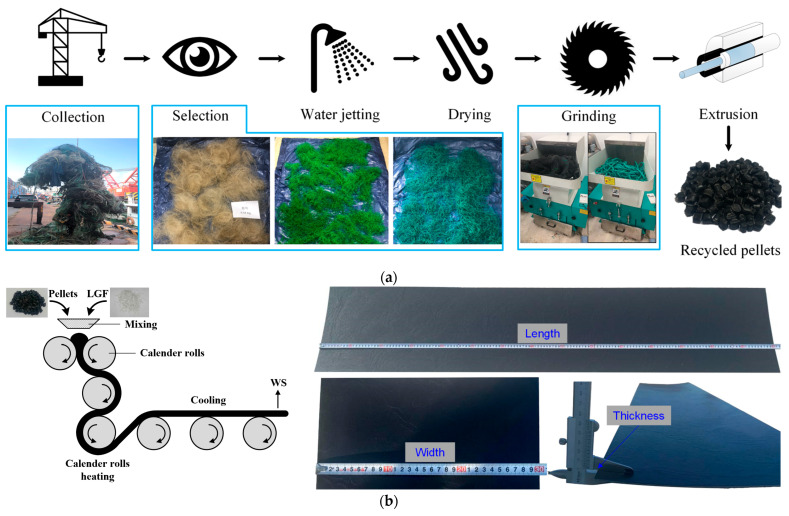
Production process of (**a**) recycled pellets and (**b**) recycled WSs.

The recycled WS utilized in this study was sourced from local fishermen at Dalpo Port in Ulsan City, Republic of Korea. The production process of recycled WSs can be divided into two stages. In the first stage, after collecting and removing unsuitable components, the WFNs undergo a thorough cleansing process by a water jet to eliminate contaminants, followed by soaking in water to remove salt residues and impurities. After air-drying at room temperature, the WFNs are shredded into flakes, which are then extruded into pellets with particle sizes ranging from 5 mm to 7 mm, as shown in Figure 1a. In the second stage, the recycled pellets are melted and combined with long glass fibers (LGFs) in a weight ratio of 0.8:0.2. The mixture is then processed through calendering to form WSs with dimensions of 1500 × 300 × 3 mm, as depicted in Figure 1b. The added LGFs, with a tensile strength of 155 MPa and a density of 1.44 g/cm^3^, were intended to enhance the tensile strength of the recycled WSs.

**Figure 2:**
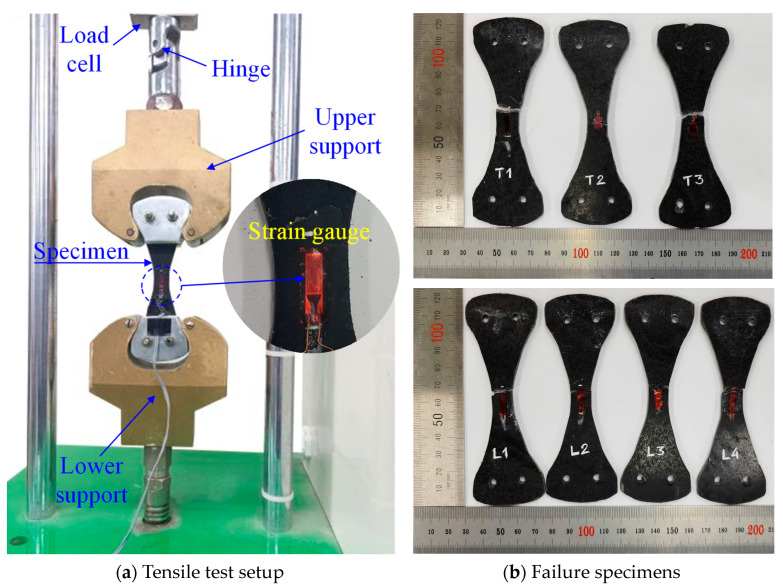
Tensile test setup and specimens.

**Figure 3:**
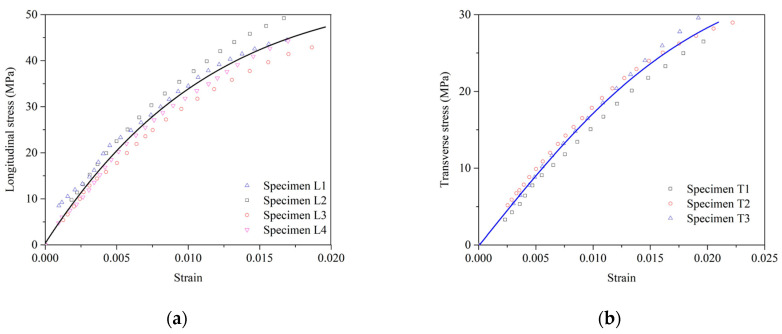
Tensile stress–strain response of a WS in the (**a**) longitudinal direction and (**b**) transverse direction.

The tensile strength of the WS was tested according to ASTM D638 [43]. The dumbbell-shaped test specimens were made to determine the longitudinal and transverse tensile properties of the WS. In Figure 2, a strain gauge measured the tensile strain of the specimens within a gauge length of 6 mm, while a load cell on the universal testing machine (UTM) recorded the applied load data. The tests were performed at a steady loading speed of 2 mm/min, utilizing a UTM with a 5 kN maximum capacity. Figure 3 presents the tensile stress–strain curve for a WS material, established using a dose–response model [44] based on experimental data from test specimens. The elastic modulus of WS was derived from the slope of the elastic stage of the longitudinal stress–strain curve. For strengthening, a commercially available two-part epoxy impregnation resin (USCHEM Co., Ltd., Seoul, Republic of Korea, EP 202) was employed to adhere the WS to the slab surface. The mechanical properties of WS and epoxy resin are given in Table 2.

### 2.2. Experimental Program

**Figure 4:**
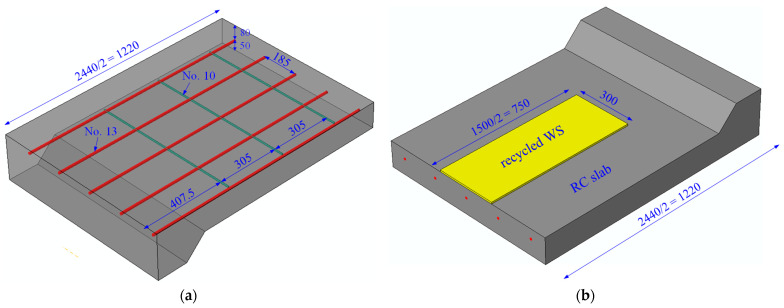
Dimensions and reinforcement details of (**a**) 1/2 control slab and (**b**) 1/2 strengthened slab (unit: mm).

An experimental program was performed to evaluate the feasibility and effectiveness of WSs in the flexural strengthening of RC slabs. Two rectangular-section RC slabs were cast for this study, one serving as a control specimen and the other strengthened using a WS. The specimens were designed with a length of 2440 mm and a clear span of 2290 mm. For the control slab, the cross-section measures 130 × 900 mm^2^, with each test specimen featuring 5 longitudinal steel bars, No.13 (ϕ12.7 mm), spaced at 185 mm intervals along the bottom side of the RC slab. Additionally, the transverse reinforcement has 6 steel bars, No.10 (ϕ9.5 mm), spaced at 305 mm intervals, as illustrated in Figure 4a. Meanwhile, the strengthened slab was fabricated by attaching a recycled WS on the underside of the RC slab, as depicted in Figure 4b.

**Figure 5:**
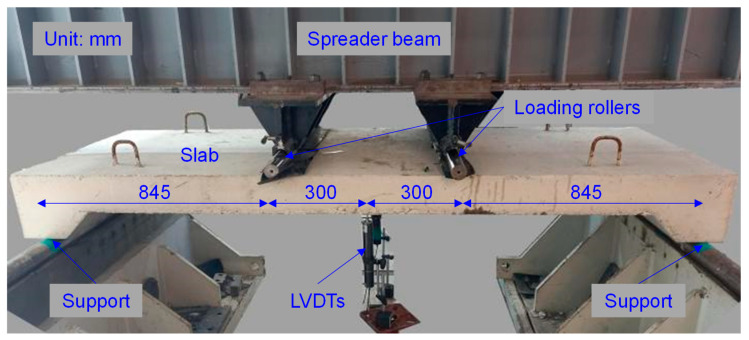
Four-point bending setup for slab.

Figure 5 shows the four-point bending test setup, with both ends supported by rigid supports and two concentrated loads symmetrically positioned 300 mm from the centerline. Deflection data at the mid-span were measured using two linear variable displacement transducers (LVDTs) with an accuracy of 0.001 mm. A load cell with a capacity of 5000 kN, integrated into the UTM, measured the applied load. The load–deflection data were continuously recorded using a calibrated computerized data acquisition system. All tests were carried out at a controlled loading rate of 5 mm/min.

### 2.3. Numerical Simulation

#### 2.3.1. Boundary Conditions, Meshing, and Element Type

**Figure 6:**
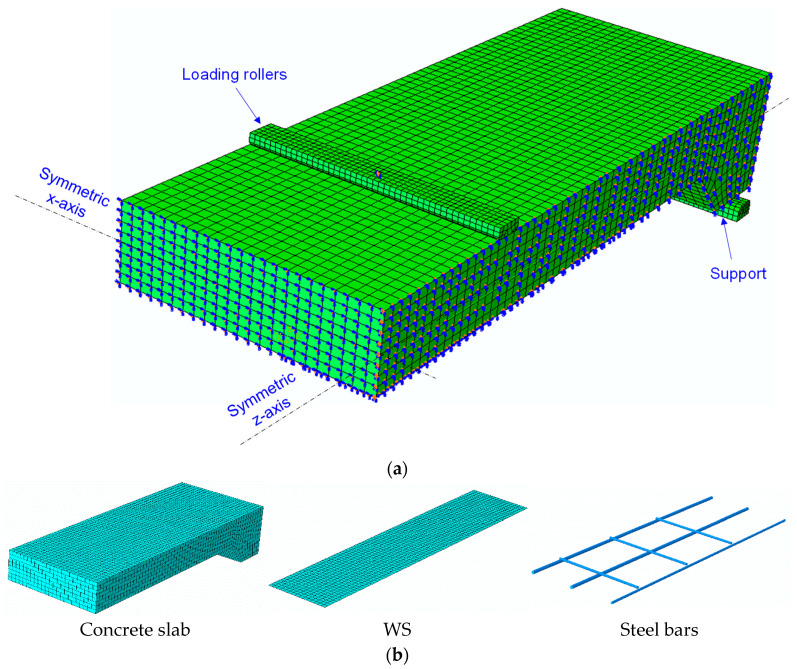
WS-strengthened slab model. (**a**) FEM meshing and boundary conditions and (**b**) elements.

The finite element analysis software ABAQUS was employed to simulate the nonlinear behavior of structural members and predict potential outcomes based on the experimental data. A model one-quarter the size of the test specimen was created by exploiting the symmetry of the specimen. Accordingly, the symmetric plane was simulated for the x- and z-axes by constraining translation in directions 1 and 3. The slab’s response, subjected to a monotonic displacement load, was further investigated under static loading conditions in a four-point bending test. The loading roller, made of a steel bar, was modeled as a rigid part to avoid stress concentrations beneath the loading point, as shown in Figure 6.

**Figure 7:**
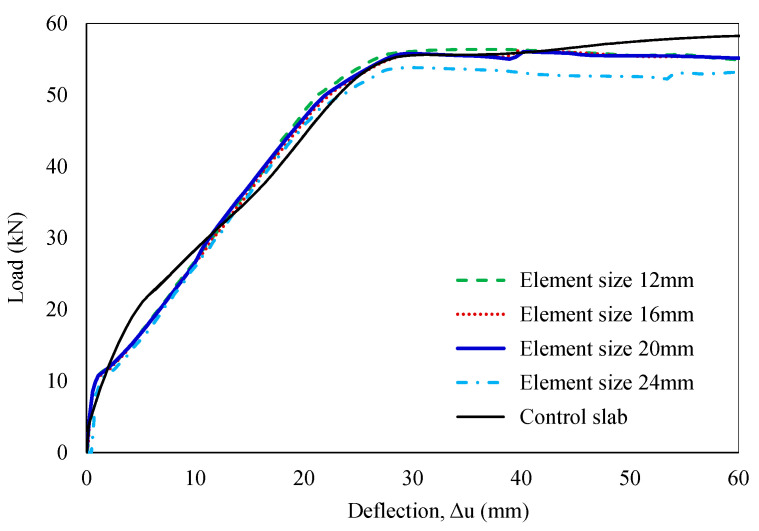
Analysis of mesh density convergence.

Achieving convergence of mesh densities is critical for the accuracy of numerical model solutions. Fine meshes can significantly increase analysis time, while coarse meshes may compromise result accuracy. Hence, four different mesh densities, ranging from 12 mm to 24 mm, were investigated in the preliminary analysis of the control slab model. A mesh size of 20 mm was recommended to obtain a balance between accuracy and computational cost. The outcomes of the mesh density investigation are shown in Figure 7. The FE model was constructed using 8-noded solid elements (C3D8R) for the concrete slab, 2-noded linear truss elements (T3D2) for the steel bars, and 4-noded shell elements (S4R) for the WS. The supports and loading rollers were modeled with 4-noded 3D rigid quadrilateral elements (R3D4), as summarized in Table 3.

#### 2.3.2. Material Model

**Figure 8:**
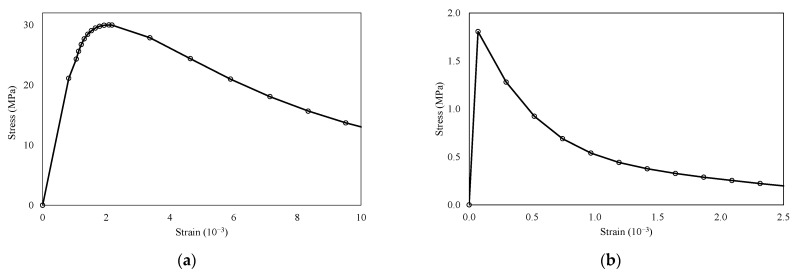
Stress–strain behavior of concrete. (**a**) Compression and (**b**) tension.

Concrete damage plasticity (CDP) is a prominent material model for plain and reinforced concrete, offering stress–strain curves that provide significant advantages compared with the available literature [45,46,47]. In this study, the behavior of reinforced concrete under static loads was simulated using the CDP model, which is highly effective for determining damage in reinforced concrete within the ABAQUS simulation software [48]. The tensile strength and Young’s modulus of concrete were determined based on the ACI 318 standard [49]. The fracture energy method was employed to characterize concrete’s post-peak tension failure behavior. The stress–strain relationship of concrete was established using the Carreira and Chu model [50], which separately considers the ascending and descending branches [51], as shown in Figure 8.

Alternatively, it was assumed that the reinforcement transmits force axially, and a widely adopted linear-elastic model for reinforcing steel was applied along with reference data from the test results of the control slab. The Poisson’s ratio of steel is a value of 0.3, and additional mechanical properties are mentioned above. In addition, the recycled WS was established in the FE model to exhibit linear and isotropic responses consistent with the experimental results discussed. Table 2 summarizes the material properties of the WS regarding thickness, elastic modulus, tensile strength, and others.

#### 2.3.3. WS-to-Concrete Interface Model

**Figure 9:**
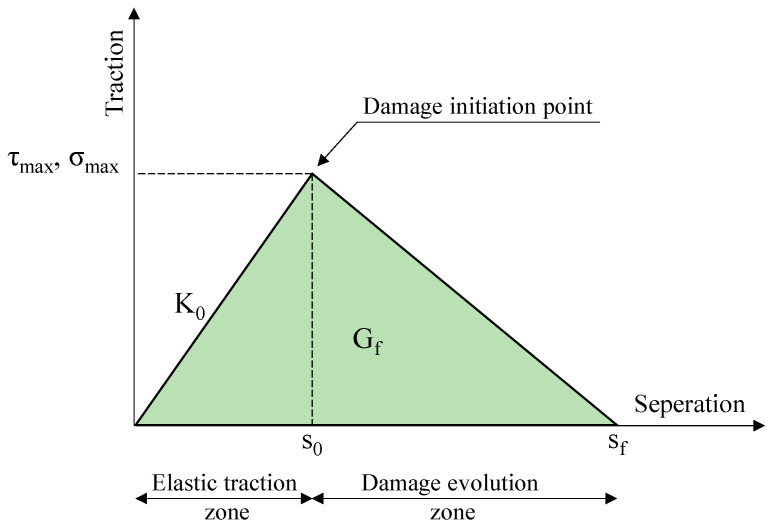
Bilinear traction–separation response.

To model the interface between the WS and the concrete slab surface, a cohesive interface was created using the traction–separation law to allow debonding failure mode. A bilinear traction–separation model was used to represent the bonding properties of the linear adhesive at the interface, as illustrated in Figure 9 [52,53,54].

As shown in Figure 9, it is evident that the traction–separation law is mainly controlled by three parameters: the interface stiffness (K_0_), maximum shear stress (τ_max_), and fracture energy (G_f_) [55]. In this study, the interaction behavior of the WS-to-concrete bond in the strengthened slab was evaluated using well-known bond–slip models, as shown in Table 4. Table 5 presents the characteristic parameters of the traction–separation law obtained from the material properties and structural dimensions investigated in this experimental work.

To define the damage initiation, quadratic nominal stress and maximum nominal stress at the WS–concrete interface were utilized. In the quadratic stress criterion, damage initiation occurs once the quadratic traction function, involving the contact stress ratios, meets the condition specified in Equation (1). Meanwhile, according to the maximum stress criterion, failure occurs upon satisfaction of the condition defined in Equation (2).

(1) $${\left(\frac{\langle \tau_{n}\rangle }{\sigma_{\max}}\right)}^{2}+{\left(\frac{\tau_{s}}{\tau_{\max}}\right)}^{2}+{\left(\frac{\tau_{t}}{\tau_{\max}}\right)}^{2}=1$$

(2) $$\max\left\{\frac{\langle \tau_{n}\rangle }{\sigma_{\max}},\frac{\tau_{s}}{\tau_{\max}},\frac{\tau_{t}}{\tau_{\max}}\right\}=1$$

In characterizing the damage evolution, the structural behavior was assessed by considering the impact of the power law (PL) and the Benzeggagh–Kenane (BK) fracture criteria. The PL criterion and BK fracture criterion can be represented by Equations (3) and (4), respectively. It was also assumed that the quantities $G_{n}^{f}$, $G_{s}^{f}$, and $G_{t}^{f}$ were equivalent in this study.

(3) $${\left(\frac{G_{n}}{G_{n}^{f}}\right)}^{\eta }+{\left(\frac{G_{s}}{G_{s}^{f}}\right)}^{\eta }+{\left(\frac{G_{t}}{G_{t}^{f}}\right)}^{\eta }=1$$

(4) $$G_{n}^{f}+(G_{s}^{f}-G_{n}^{f}){\left(\frac{G_{s}+G_{t}}{G_{n}+G_{s}}\right)}^{\eta }=G_{f}$$

#### 2.3.4. Analysis Process

**Figure 10:**
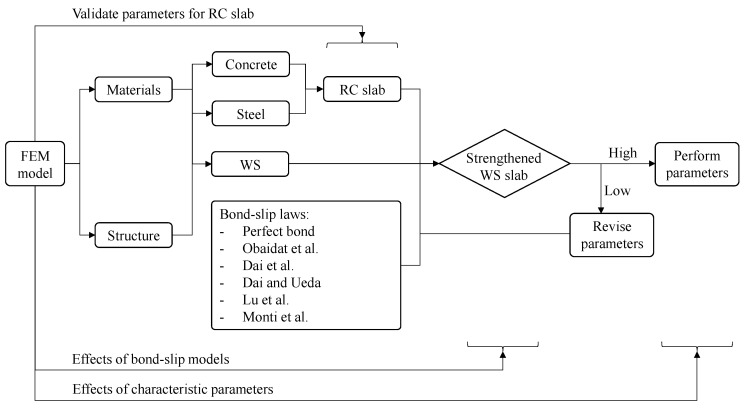
Diagram analyzing the impact of bond–slip parameters on debonding behavior [34,56,57,58,59].

An analysis was conducted using FE modeling to simulate the behavior of a slab strengthened with recycled WS. First, an FE model of the control slab was validated against experimental results to ensure the proper values of input parameters for the RC slab model. Next, a strengthened slab was simulated by attaching a WS to the bottom side of the RC slab model. A perfect bond model and renowned bond–slip models were also used to analyze the WS-to-concrete interface and their effect on the debonding failure of the strengthened slab. Finally, adjustments to the characteristic parameters could lead to a good agreement with the experimental results, enabling an evaluation of their influence on the recycled WS-to-concrete interface. A diagram analyzing the impact of the characteristic parameters on debonding behavior is shown in Figure 10.

## 3. Results and Discussion

### 3.1. Experimental Analysis

**Figure 11:**
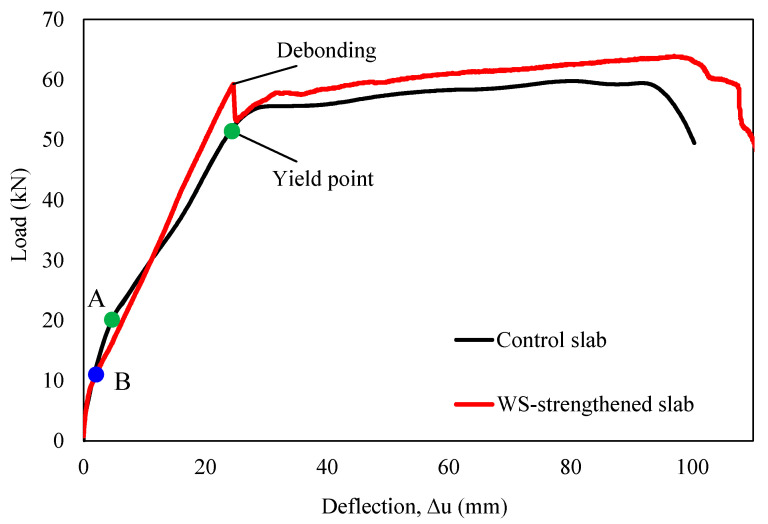
Load–deflection relationship for the control and strengthened slabs.

**Figure 12:**
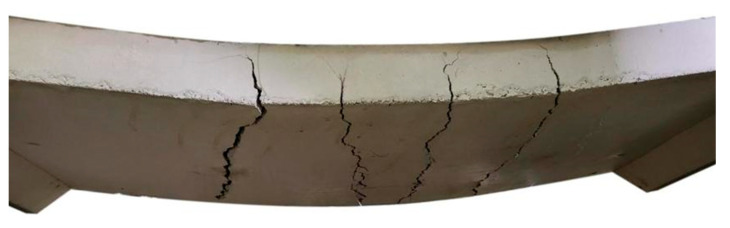
Cracks and the failure mode of the control slab.

Table 6 summarizes the ultimate load, mid-span deflection, and initial stiffness for both the control and strengthened slab, whereas Figure 11 depicts their load–deflection (P−Δ) relationship. Figure 12 illustrates the failure mode of the control slab. In this test, the RC slab exhibited a linear-elastic response until microcracks emerged at an applied load of 18.9 kN on the bottom side near the mid-span section, as depicted by point “A” in Figure 11, marking the initial slope’s change. Afterward, the control slab maintained a linear-elastic behavior as the reinforcement reached the yield state at an applied load of 51.4 kN with a mid-span deflection of 24.1 mm. The ultimate load of the control slab was recorded as 59.7 kN, corresponding to a mid-span deflection of 80.5 mm. The slope of the P−Δ curve in the linear elastic stage can be utilized to analyze the initial stiffness [60]. In this case, the initial stiffness of the specimen was calculated as 2.31 kN/mm.

**Figure 13:**
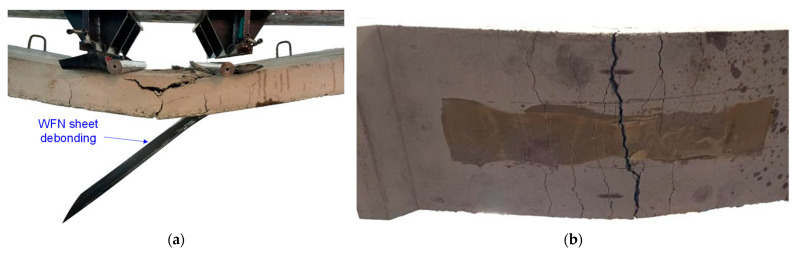
Cracks and the failure mode of slab strengthened by recycled WS. (**a**) Front view and (**b**) bottom view.

For the strengthened slab, it was observed that microcracks developed at a lower load (11 kN) compared to the control slab, as indicated by the change in slope at point B in Figure 11. It may be attributed to the limited area covered by the reinforcing layer (one-third of the slab width) and potential inconsistencies in material composition between specimens. Nevertheless, this discrepancy did not significantly influence its global response and load-carrying capacity. The strengthened slab exhibited linear-elastic behavior up to an applied load of 59.3 kN. Debonding failure then occurred, with a sudden load drop to 53.3 kN (an 11.3% decrease) at a mid-span deflection of 24.6 mm. After debonding, the global response of the slab matched that of the control slab, reaching the ultimate load of 63.9 kN at a mid-span deflection of 97 mm. The strengthened slab exhibited a 13% increase in initial stiffness compared to the control slab, reaching a value of 2.41 kN/mm. Remarkably, the strengthened slab maintained a linear-elastic response until WS delamination, increasing the yield load of the strengthened slabs relative to that of the control by 15%. It validated the feasibility of using recycled WSs for strengthening RC slabs. In addition, the debonding load was less than the ultimate load, and the WS did not experience significant damage, as shown in Figure 13. It indicates that the debonding failure of the WS from the concrete substrate reduced the load-carrying capacity of the reinforced element. It also confirms that using epoxy resin alone to bond these two materials might not maximize the strengthening effect. It is worth noting that the proportion of WS used is relatively small, offering an opportunity for optimization; the ultimate failure load will increase with increasing the area and number of layers of WSs, but is not proportional. 

### 3.2. Numerical Bond–Slip Analysis

#### 3.2.1. Effect of Bond–Slip Models

The FE model of the strengthened slab was developed to investigate the global response under a quasi-static load following the successful validation of the control slab simulation results with experimental data. A perfect bond and renowned bond–slip models, utilizing the characteristic parameters detailed in Table 5, were employed to simulate the strengthened slab response involving failure modes and the load–deflection relationship. The quadratic nominal stress criterion is applied to define the damage initiation at the interface between WS and concrete. Meanwhile, the BK law fracture criterion, featuring a cohesive coefficient set at one (η = 1), is utilized to depict the dependency of fracture energy on both opening and sliding failure modes. The FE model employed a simplified approach to post-debonding behavior to achieve computational efficiency and reduce processing time and costs. It was developed to accurately simulate the response of the slabs up to the yielding phase in the reinforcement after debonding, rather than modeling the entire response during testing.

**Figure 14:**
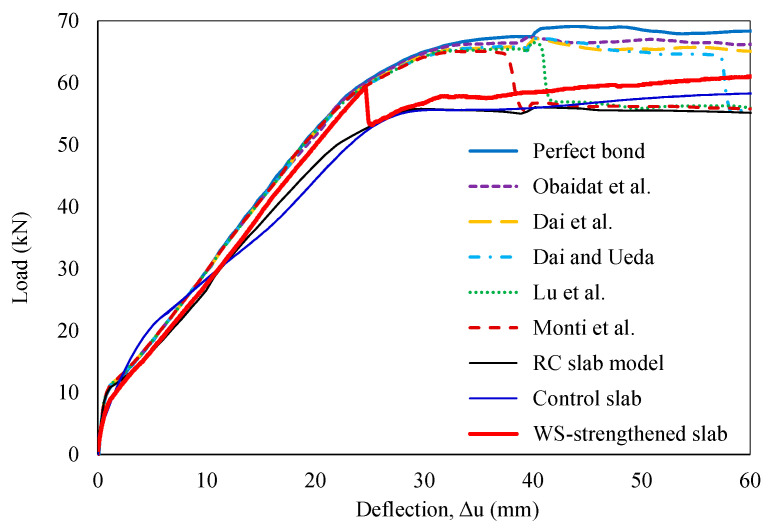
FEM predictions of load–deflection relationship [34,56,57,58,59].

In Figure 14, the FE model, incorporating the specified bond–slip laws, accurately captured the initial linear stage of the experimental P−Δ curve. However, it revealed limitations in predicting the debonding behavior observed in the experiments. The bond–slip models proposed by Monti et al., Lu et al., and Dai and Ueda predicted debonding failure at mid-span deflections of 37.8 mm, 40 mm, and 57 mm, respectively. Conversely, the remaining models maintained an idealized perfect bond until the analysis was discontinued at a mid-span deflection of 60 mm. Most existing bond–slip models could predict the yield load and corresponding mid-span deflection with errors of less than 2%, resulting in negligible discrepancies in initial stiffness. However, relatively large errors were observed for the debonding load, ranging from 9% to 12%, or debonding failure was not predicted within the analysis range. More significantly, these models struggled to accurately predict mid-span deflections at debonding, with errors exceeding 54%, as summarized in Table 7. Overall, these models predicted a higher bond strength between the WS and the concrete substrate than the experimental data. The observed discrepancies may stem from inaccurate estimations of the bond–slip characteristic parameters involving fracture energy, interface stiffness, maximum shear stress, damage initiation criteria, or mixed-mode failure behavior.

#### 3.2.2. Effect of Characteristic Parameters

**Figure 15:**
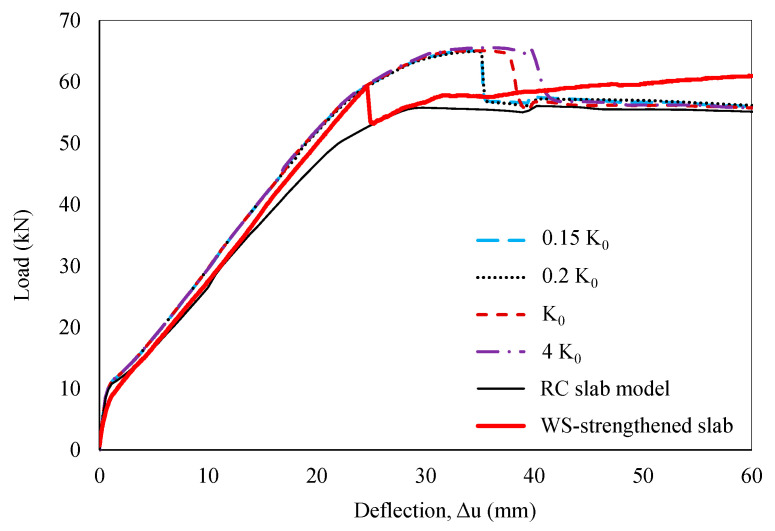
Sensitivity analysis of interface stiffness.

The mentioned bond–slip models overestimated the bond strength of the WS-to-concrete interface. Hence, further research on the influence of the related characteristic parameters for the WS-to-concrete interface is essential to propose an appropriate bond–slip law for these materials. A sensitivity analysis of the input characteristic parameters was carried out using FE models to assess their impact on the structural response under quasi-static loading conditions. The bond–slip parameters proposed by Monti et al. [59] were employed to evaluate the obtained numerical data for illustration purposes. Based on the experimental results, the numerical analysis was terminated at a mid-span deflection of 60 mm to focus on the effects of characteristic parameters on debonding behavior and reduce processing time. To encompass a broader range of interfacial behavior relevant to potential bond–slip models, the influence of K_0_, ranging from 0.15K_0_ to 4K_0_, was explored, as shown in Figure 15. While the P–Δ curve’s slope exhibited minimal sensitivity to initial stiffness, the behavior of debonding failure was considerably affected. Debonding occurred earlier at lower K_0_ values (0.15K_0_ and 0.2K_0_) and later at higher values (4K_0_). The findings indicate that the debonding load remains relatively constant at 65.4 kN for interface stiffnesses ranging from 0.15K_0_ to 4K_0_, with corresponding mid-span deflections varying from 34.9 mm to 39.8 mm. Notably, the global model response remained consistent at or below 0.2K_0_, yet it still overestimated the interfacial bond strength between the WS and the concrete substrate.

**Figure 16:**
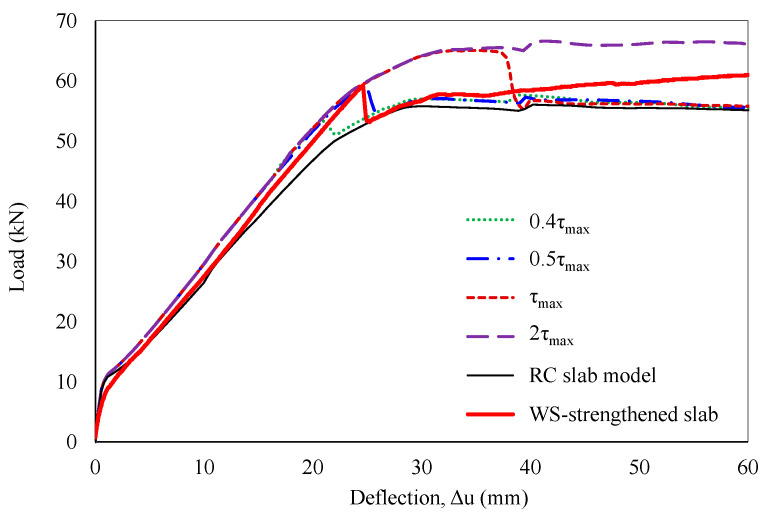
Sensitivity analysis of maximum shear stress.

In addition, a sensitivity analysis of maximum interfacial shear stress, ranging from 0.4τ_max_ to 2τ_max_, was performed to investigate its impact on debonding behavior. The results indicated a strong correlation between maximum shear stress and debonding failure, with earlier debonding observed at lower τ_max_ values. Debonding occurred at loads of 52.9 kN, 59 kN, and 65.4 kN, with corresponding mid-span deflections of 21.4 mm, 24.7 mm, and 37.8 mm, at maximum shear stresses of 0.4τ_max_, 0.5τ_max_, and τ_max_, respectively. For maximum shear stress exceeding 2τ_max_, an idealized perfect bond was predicted. As a result, a maximum shear stress of 0.5τ_max_ yielded a relatively accurate simulation of the strengthened slab’s global response, as shown in Figure 16.

**Figure 17:**
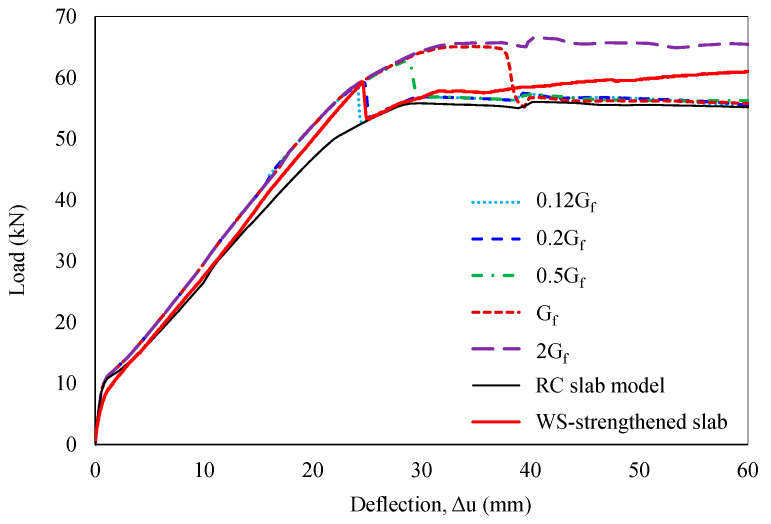
Sensitivity analysis of fracture energy.

The influence of another characteristic parameter—interfacial fracture energy—was also further investigated, considering values from 0.12G_f_ to 2G_f_. The debonding failure emerged earlier with decreasing G_f_ and vice versa, demonstrating a nonlinear relationship. Sensitivity to fracture energy was particularly evident within the 0.12G_f_ to G_f_ range, with debonding loads varying from 58.6 kN to 65.4 kN and corresponding mid-span deflections ranging from 24.1 mm to 37.8 mm. A perfect bond was predicted for interfacial fracture energy values greater than or equal to 2G_f_. Remarkably, a fracture energy value of 0.2G_f_ enabled a reasonable simulation of the WS-strengthened slab’s behavior, as shown in Figure 17.

**Figure 18:**
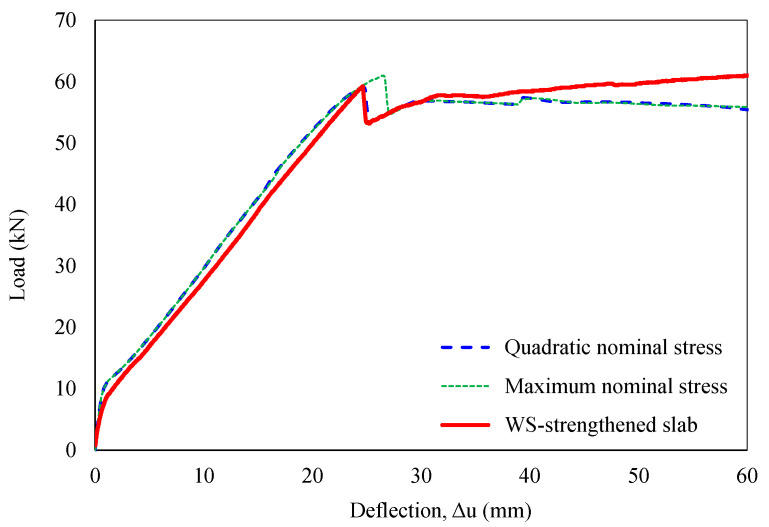
Sensitivity analysis of damage initiation criteria.

Based on the obtained results, this section analyzes two damage initiation criteria: quadratic nominal and maximum nominal stress. The quadratic nominal stress criterion considers the combined influence of all three traction components, while the maximum nominal stress criterion compares each component’s stress to its respective limit value [61]. As shown in Figure 18, the quadratic and maximum nominal stress criteria showed different mid-span deflections at 24.6 mm and 26.5 mm at debonding failures, respectively. Although consistent with the previous finding [30], the effect of damage initiation criteria on the strengthened slab’s response was less pronounced in this study. This discrepancy can be attributed to the smaller strengthened area (one-third of the slab width) compared to full-width strengthening employed in previous work. Accordingly, a significant influence of all stress components was recognized for decohesive elements during mixed-mode delamination initiation and propagation, consistent with the findings of Cui et al. [62]. Debonding could occur before any individual traction component reaches its allowable limit, with this phenomenon becoming more pronounced in wider strengthened sections.

**Figure 19:**
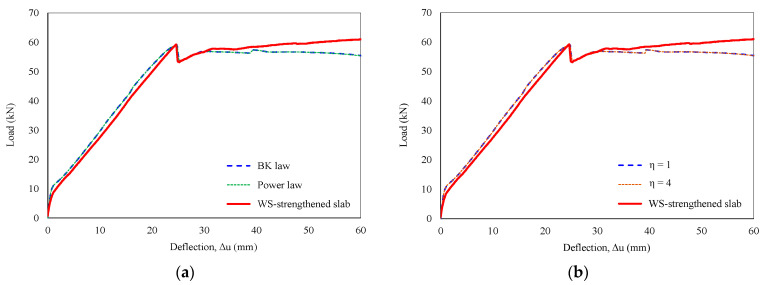
Sensitivity analysis of characteristic parameters. (**a**) Fracture criteria and (**b**) cohesive coefficient.

Furthermore, the influence of fracture criteria under mixed-mode conditions (BK law and power law) on the P–Δ curve is explored in Figure 19a, along with a sensitivity analysis of the cohesive coefficient in Figure 19b. Notably, these parameters exhibited minimal impact on the debonding failure or the overall response of the slab.

In summary, the characteristic parameters K_0_, τ_max_, and G_f_ significantly influence the global response and, more specifically, the debonding behavior of strengthened slabs. Accurate prediction of WS-strengthened slab behavior requires careful consideration of these parameters, along with the interaction between stress components. Moreover, the importance of these parameters for predicting the behavior of broader classes of externally bonded FRP- or WS-strengthened concrete structures is highlighted.

## 4. Conclusions

This study investigated the application of recycled WSs as a sustainable material for strengthening existing RC slabs. The global behavior of the RC slabs with and without strengthening was presented. The impact of existing bond–slip models and characteristic parameters on the debonding failure was evaluated. The conclusions based on the main findings could be summarized as follows:

The recycled WS significantly enhances the RC slab capacities, resulting in a 15% higher yield load and a 13% increase in initial stiffness for the strengthened structure.

Existing bond–slip models overestimated bond strength at the WS-to-concrete substrate interface. The numerical analysis, employing a maximum shear stress of 0.5τ_max_ or an interfacial fracture energy of 0.2G_f_ compared to their counterpart values proposed by Monti et al., demonstrated a strong correlation with the experimental data.

The sensitivity of strengthened slab behavior to damage initiation criteria was found to be significantly influenced by the dimensions of the strengthened section. Conversely, damage evolution criteria involving mixed-mode failure and cohesive coefficients exhibited minimal impact on the overall structural response.

The WSs, recycled from WFNs, offer a sustainable solution for rehabilitating damaged RC structures, especially in marine constructions prone to chloride corrosion. Further research is recommended to explore methods for enhancing adhesion by combining epoxy resin with steel anchors. Additionally, addressing practical challenges such as WFN collection and pretreatment, and optimizing the recycling process is essential.

## Figures and Tables

**Table 1:** The ratio of components and the compressive strength of concrete by weight.

Cement	Coarse Aggregate	Fine Aggregate	Water	f’_c_ (MPa)
1	3.18	1.56	0.52	30

**Table 2:** Mechanical properties of WS and epoxy resin.

		WS					Epoxy	
$f_{\mathrm{WS}}^{l}$ (MPa)	$\varepsilon_{\mathrm{WS}}^{l}$	$f_{\mathrm{WS}}^{t}$ (MPa)	$\varepsilon_{\mathrm{WS}}^{t}$	$E_{\mathrm{WS}}$ (MPa)	$\rho_{\mathrm{WS}}$ (kg/m^3^)	t_a_ (mm)	E_a_ (MPa)	G_a_ (MPa)
45	0.0183	29	0.0210	4200	980	1.5	4500	2100

**Table 3:** Element types for model members in the ABAQUS software.

Notation	Element Type
Concrete slab	C3D8R
Steel bar	T3D2
WS	S4R
Support, loading roller	R3D4

**Table 4:** Existing bond–slip models.

Constitutive Model	Interface Stiffness (K_0_)	Maximum Shear Stress (τ_max_)	Fracture Energy (G_f_)	Notation
Obaidat et al. [34]	$0.16\frac{G_{a}}{t_{a}}+0.47$	$1.46{\;G}_{a}^{0.165}f_{\mathrm{ct}}^{1.033}$	$0{.52\;f}_{\mathrm{ct}}^{0.26}G_{a}^{-0.23}$	
Dai et al. [56]	$40.08\;{\left(f{'}_{c}\right)}^{0.236}$	$2.67\;{\left(f{'}_{c}\right)}^{0.236}$	$0.514\;{\left(f{'}_{c}\right)}^{0.236}$	
Dai and Ueda [57]	${\alpha \;K}_{a}$	$\frac{-1.575{\alpha K}_{a}+\sqrt{2.481\alpha^{2}K_{a}^{2}+6.3\alpha \beta^{2}K_{a}G_{f}}}{2\beta }$	$7.554{\;K}_{a}^{-0.449}{\left(f{'}_{c}\right)}^{0.343}$	$\begin{array}{l} \beta =0.0035K_{a}{(E_{f}t_{f}/1000)}^{0.34}\\ \alpha =0.028{\left(E_{f}t_{f}/1000\right)}^{0.254}\\ K_{a}=G_{a}/t_{a} \end{array}$
Lu et al. [58]	76.92	$1{.5\;\beta }_{w}f_{\mathrm{ct}}$	$0.308\;\beta_{w}^{2}\sqrt{f_{\mathrm{ct}}}$	$\beta_{w}=\sqrt{\frac{2.25-b_{f}{/b}_{c}}{1.25+b_{f}{/b}_{c}}}$
Monti et al. [59]	${\left[2.5\left(\frac{t_{a}}{E_{a}}+\frac{50}{E_{c}}\right)\right]}^{-1}$	$1.8{\;k}_{b}f_{\mathrm{ct}}$	$0.297{\;k}_{b}^{2}f_{\mathrm{ct}}$	$k_{b}=\sqrt{\frac{1.5(2-b_{f}{/b}_{c})}{1+b_{f}/100\mathrm{mm}}}$

**Table 5:** Characteristic parameters of bond–slip models.

Constitutive Model	K_0_ (MPa/mm)	τ_max_ (MPa)	G_f_ (N/mm)
Obaidat et al. [34]	224.47	18.26	0.12
Dai et al. [56]	89.44	5.96	1.15
Dai and Ueda [57]	74.61	6.59	0.94
Lu et al. [58]	76.92	5.61	0.69
Monti et al. [59]	163.78	4.84	0.63

**Table 6:** Summary of experimental data.

Notation	P_y,exp._ (kN)	Δ_y,exp._ (mm)	P_u,exp._ (kN)	Δ_u,exp._ (mm)	P_d,exp._ (kN)	Δ_d,exp._ (mm)	K_exp._ (kN/mm)
Control slab	51.4	24.1	59.7	80.5	-	-	2.13
Strengthened slab	59.3	24.6	63.9	97.0	59.3	24.6	2.41

**Table 7:** FE analysis for strengthened slab compared with experimental data.

Constitutive Models	P_y,mod._ (kN)	Δ_y,mod._ (mm)	K_mod._ (kN/mm)	P_d,mod._ (kN)	Δ_d,mod._ (mm)	$\frac{P_{y,mod.}}{P_{y,exp.}}$	$\frac{{∆}_{y,mod.}}{{∆}_{y,exp.}}$	$\frac{K_{mod.}}{K_{exp.}}$	$\frac{P_{d,mod.}}{P_{d,exp.}}$	$\frac{{∆}_{d,mod.}}{{∆}_{d,exp.}}$
Monti et al. [59]	59.4	24.6	2.41	65.4	37.8	1.00	1.00	1.00	1.10	1.54
Lu et al. [58]	59.6	24.7	2.41	66.5	40.0	1.01	1.00	1.00	1.12	1.63
Dai and Ueda [57]	59.6	24.8	2.40	64.4	57.0	1.01	1.01	1.00	1.09	2.32
Dai et al. [56]	59.1	24.2	2.44	-	-	1.00	0.98	1.01	-	-
Obaidat et al. [34]	60.4	25.0	2.42	-	-	1.02	1.02	1.00	-	-
Perfect bond	60.4	24.8	2.44	-	-	1.02	1.01	1.01	-	-

## Data Availability

The original contributions presented in the study are included in the article, further inquiries can be directed to the corresponding author.

## Nomenclature

b_c_	Width of RC slab
b_F_	Width of FRP sheet
E_a_	Tensile modulus of elasticity of epoxy resin
E_c_	Modulus of elasticity of concrete
E_F_	Tensile modulus of elasticity of FRP
E_WS_	Tensile modulus of elasticity of WS
f’_c_	Compressive strength of concrete
f_ct_	Tensile strength of concrete
$f_{WS}^{l}$	Longitudinal tensile strength of WS
$f_{WS}^{t}$	Transverse tensile strength of WS
G_a_	Shear modulus of epoxy resin
G_f_	Fracture energy
G_n,_ G_s,_ G_t_	Fracture energies in the normal, first, and second shear directions
$G_{n}^{f},G_{s}^{f},G_{t}^{f}$	Fracture energies to induce failure in the normal, first, and second shear directions
K_0_	Interface stiffness
K_exp._, K_mod._	Initial stiffness in the experimental and numerical analysis
P_y.exp._, P_u.exp._, P_d.exp._	Yieding load, ultimate load, and debonding load in the experiment
P_y.mod._, P_u.mod._, P_d.mod._	Yieding load, ultimate load, and debonding load in the numerical analysis
s_f_	Maximum local slip
s_0_	Local slip at maximum shear stress
t_a_	Thickness of epoxy resin
t_F_	Thickness of FRP laminate
τ	Shear stress
τ_max_	Maximum shear stress
$\tau_{n},\tau_{s},\tau_{t}$	Normal, first, and second shear stress
σ_max_	Maximum nominal stress
η	Cohesive coefficient
α	Parameter in bond–slip relationships
β_w_	Width ratio factor
γ_c_	Specific gravity of concrete
$\rho_{WS}$	Unit weight of WS
$\varepsilon_{WS}^{l}$	Longitudinal ultimate strain of WS
$\varepsilon_{WS}^{t}$	Transverse ultimate strain of WS
Δ_y,exp._, Δ_u,exp._, Δ_d,exp._	Mid-span deflection at yielding load, ultimate load, and debonding load in the experiment
Δ_y,mod._, Δ_u,mod._, Δ_d,mod._	Mid-span deflection at yielding load, ultimate load, and debonding load in the numerical analysis
⟨ ⟩	Macaulay brackets, indicating that compressive stress does not cause initial damage
